# The implications of thumb movements for Neanderthal and modern human manipulation

**DOI:** 10.1038/s41598-020-75694-2

**Published:** 2020-11-26

**Authors:** Ameline Bardo, Marie-Hélène Moncel, Christopher J. Dunmore, Tracy L. Kivell, Emmanuelle Pouydebat, Raphaël Cornette

**Affiliations:** 1 Skeletal Biology Research Centre, School of Anthropology and Conservation, University of Kent, Canterbury, Kent, CT2 7NR UK; 2 Département Homme et environnement, UMR7194–HNHP (CNRS – MNHN – UPVD – Sorbonne Universités), 1 rue René-Panhard, 75005 Paris, France; 3 Department of Human Evolution, Max Planck Institute for Evolutionary Anthropology, 04103 Leipzig, Germany; 4 UMR 7179 Mécanismes Adaptatifs et Evolution (CNRS, MNHN), Muséum national d’Histoire naturelle, 55 rue Buffon, 75005 Paris, France; 5 Institut de Systématique, Evolution, Biodiversité (ISYEB), Muséum national d’Histoire naturelle, CNRS, Sorbonne Université, EPHE, Université des Antilles, CP 50, 57 rue Cuvier, 75005 Paris, France

**Keywords:** Evolution, Anthropology, Cultural evolution

## Abstract

Much research has debated the technological abilities of Neanderthals relative to those of early modern humans, with a particular focus on subtle differences in thumb morphology and how this may reflect differences in manipulative behaviors in these two species. Here, we provide a novel perspective on this debate through a 3D geometric morphometric analysis of shape covariation between the trapezial and proximal first metacarpal articular surfaces of Neanderthals (*Homo neanderthalensis*) in comparison to early and recent humans (*Homo sapiens*). Results show a distinct pattern of shape covariation in Neanderthals, consistent with more extended and adducted thumb postures that may reflect habitual use of grips commonly used for hafted tools. Both Neanderthals and recent humans demonstrate high intraspecific variation in shape covariation. This intraspecific variation is likely the result of genetic and/or developmental differences, but may also reflect, in part, differing functional requirements imposed by the use of varied tool-kits. These results underscore the importance of holistic joint shape analysis for understanding the functional capabilities and evolution of the modern human thumb.

## Introduction

Variation in fossil hominin hand morphology has played a key role in the interpretation of how human manipulative abilities evolved^1–5^. There has been a particular focus on the thumb and the radial wrist bones, as their morphology is thought to reflect interspecific differences in the frequency and complexity of tool-related behaviors^2–15^. To better understand the morphological transitions that lead to the anatomically modern human (*Homo sapiens*) hand, many studies have analyzed how the human hand differs from that of Neanderthals (*Homo neanderthalensis*)^4,11–13,16^. Morpho-functional interpretations generally agree that both modern humans and Neanderthals were likely capable of the same dexterity^4,17^. However, based on their robust phalanges, broader distal phalanges and joint configurations (see below), Neanderthal hands appear better adapted for forceful power grips that are considered important for the effective use of some tools, such as hafted Mousterian spears and scrapers^11,13,17–20^. However, a recent study by Karakostis et al.^16^ argued that Neanderthal hand muscle attachment areas (entheses) are similar to those of recent humans that used precision grips throughout their professional life (e.g., tailors, shoemakers, joiners), suggesting the use of habitual precision, rather than power, grasping in Neanderthals. To better understand how Neanderthal and modern human thumb function may have varied, it is important to evaluate how the joints of the trapezium (including the first and second metacarpals, trapezoid and scaphoid facets) and the proximal joint of the first metacarpal (Mc1) correspond to each other. These joints are the primary osteological determinant of thumb mobility^21^ and we refer to all of these joints together as the trapeziometacarpal (TMc) complex. Building on previous work^4,11,12,18^, we investigate the morpho-functional characteristics of the thumb in Neanderthals, as well as early and recent modern humans, through a three-dimensional (3D) geometric morphometric (GM) analysis^22^ of shape covariation between the joints of the TMc complex. This analysis of the entire trapeziometacarpal anatomical region is a more holistic approach than in previous studies that have only focused on the trapezium-Mc1 articulation or these bones in isolation^7,8,10,12,14,15^.

The morphological configuration of the thumb and radial side of the wrist is broadly similar between the modern human and Neanderthal hands^5^. Compared with other great apes, as well as some fossil hominins^23,24^, modern humans and Neanderthals both show a broad, relatively flat trapezial–metacarpal joint, including a palmarly-expanded trapezoid and an extensive trapezium-scaphoid joint. Together, these features have been interpreted as biomechanically advantageous for high loading from the thumb during frequent tool use and production^3,6–8^. However, the biomechanical implications of subtle morphological differences between the TMc complexes of Neanderthals and modern humans have been less clear^18^. Compared with modern humans, Neanderthals have a larger trapezial-Mc1 joint area that is dorsopalmarly flatter^10,12,13,18^. This joint morphology has been interpreted as less congruent and, therefore, possessing greater mobility that, in turn, would require greater muscular force, or ligamentous support, than that of modern humans to achieve the same level of joint stability^18^. Combined with other features of the hand, including robust phalanges, rugose musculotendinous attachment sites, more parasagittally-oriented capitate-second metacarpal facets, reduced third metacarpal styloid processes, radioulnarly flat fifth metacarpal bases, and large, projecting carpal tubercles, this trapezial-Mc1 joint morphology has been interpreted as evidence that power grips may have been more frequently used in the Neanderthal manipulative repertoire than that of early modern humans^12,19,20^. However, there is considerable intraspecific variation in Neanderthal trapezial-Mc1 joint shape, and some specimens (e.g., La Ferrassie 1) are difficult to distinguish from recent humans. Together with notable morphological variation in the TMc complex morphology overall (e.g. Kebara 2)^19,20^, this morphology makes characterizing a ‘typical’ Neanderthal morphology challenging. An analysis of shape covariation across the TMc complex may shed light on the subtle functional consequences of this morphological variation within different Neanderthal individuals. Neanderthals had tool-kits comprising diverse lithic types and sizes^25^ that would require different hand grips to use^26^, but Neanderthals may also have practiced varied grasping behaviours due to differences in geography^27^, activities, time^28^ and/or sex^29^, all of which could be reflected within hand morphology.

**Figure 1:**
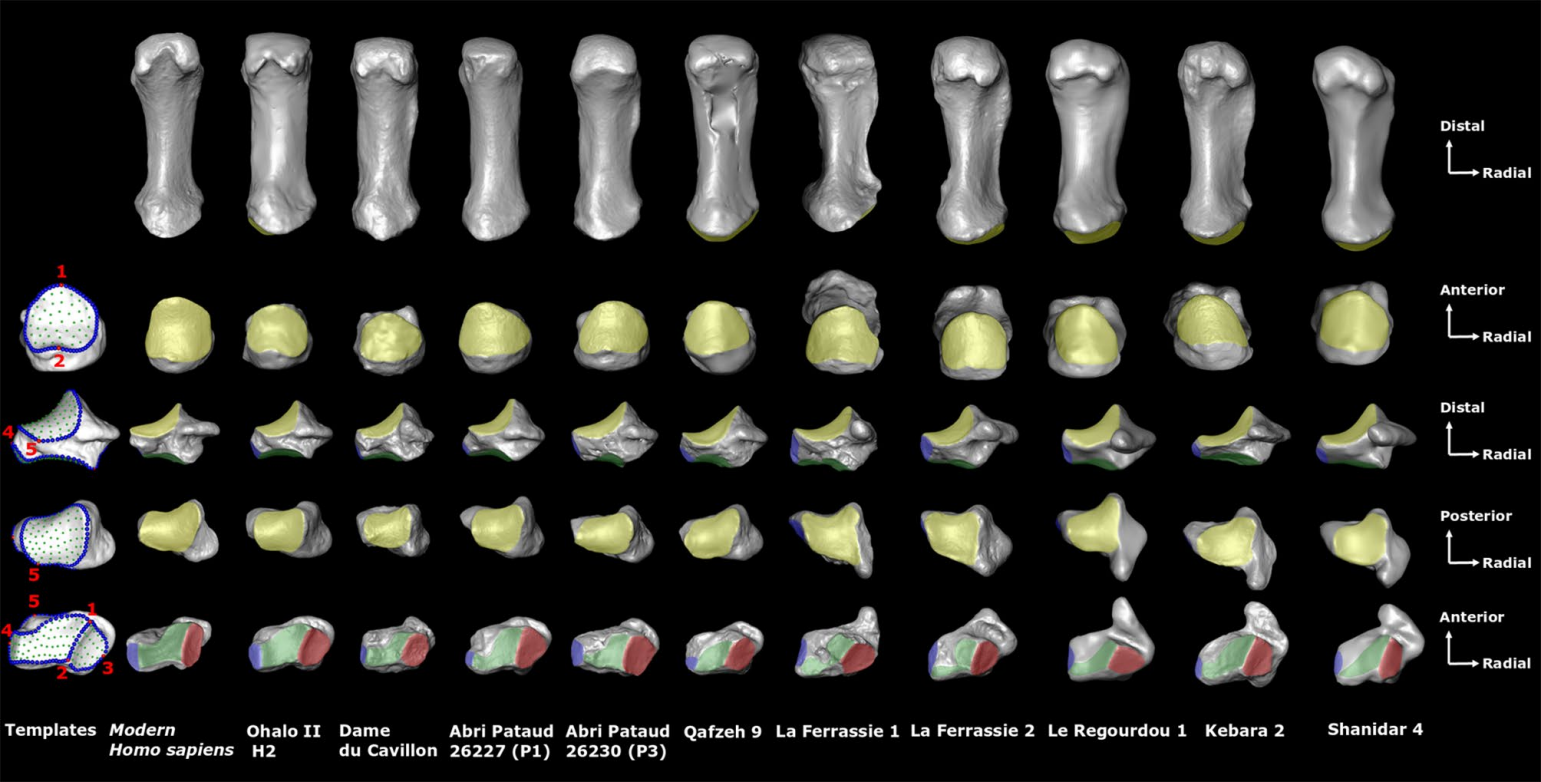
Joint shape comparison of the Mc1 (top 1st row, palmar view; top 2nd row, proximal view) and trapezium (middle row, palmar view; 1st row from bottom, proximal view; 2nd row from bottom, distal view) in modern human (2nd from left) and five early humans (3rd to 7th from the left) and five Neanderthals (1st to 5th from right). Key colors: yellow, trapezial-Mc1 joint; blue, 2nd metacarpal joint; green, trapezoid joint; red, scaphoid joint. The first column (left) represents the landmark templates used in our analyses to quantify shape covariation (see “Materials and methods” section, and detailed in Supplementary Information Figure S1 and Table S3). The illustration is not scaled, and bones from the left-hand side (Le Régourdou 1, Kebara 2, Shanidar 4, Abri Pataud P1, Abri Pataud P3, Dame du Cavillon) are mirrored for fair comparison.

The shape variation found in previous studies in Neanderthals and modern humans^7,8,10–15,20^, may be the result of multiple of factors, including genetics, evolutionary history, hormones, sex, geography, and common developmental origin^30^. However, since bone (re)models throughout life, it may also reflect, in part, variation in habitual use of the hand during ontogeny. Although joint shape is commonly considered to be more genetically and functionally constrained than other aspects of bone shape (e.g., shaft external or internal bone structure)^31,32^, within the hand, and in particular the small bones of the carpus, the constraints on joint shape are less clear. The trapezium does not complete ossification in humans until 9–10 years of age^33^, while the base of the Mc1 does not completely fuse until 14–16.5 years of age. The trapezium develops within the hand surrounded by, and incurring load from, five other bones. Further, both the trapezium and Mc1 will incur substantial muscular force, directly or indirectly, from the intrinsic and extrinsic muscles of the radial side of the hand. Strong and complex manipulative abilities are observed in modern humans before the end of the total ossification of their carpal bones and the Mc1^34^. Furthermore, Neanderthals are thought to have made and used tools as juveniles^35^. As such, it is possible that frequent loading from habitual manual activities during development and adulthood may subtly affect how the bones of the TMc complex articulate with each other as their joint surfaces ossify. In this study, we assess the morphological variation in the associated trapezia and first metacarpals of five Neanderthal individuals (La Ferrassie 1 and 2, Le Régourdou 1, Kebara 2, Shanidar 4) and compare them to five early modern humans (Qafzeh 9, Ohalo 2, Abri Pataud 26227 (AP-P1), Abri Pataud 26230 (AP-P3), Dame du Cavillon) as well as a broad sample of recent humans (Table 1; Fig. 1, and Supplementary Information Table S1). Through a 3D GM approach using sliding semi-landmarks^22^, we analyze shape covariation across the joints of the TMc complex. While previous analyses of 3D shape variation in the isolated trapezium, Mc1 or trapezoid have revealed interspecific differences across hominins species^7,8,14,15^, the movement and loading of the thumb is largely delimited by the interaction of the bones of the TMc complex together. By analyzing shape covariation, we quantify, for the first time, how joint shapes vary together across the trapezium and Mc1. That is, we explore how change in articular shape of each articular facet is reflected in the shape of the remaining TMc complex facets. Just as the functional interaction of the trapezial-Mc1 joint is the primary osteological determinant of thumb mobility^21^, we assume that all the functional joints of the two bones covary to some extent. We aim to test the null hypothesis that joints of the TMc complex covary in the same way (i.e., same shape and relative orientations of the TMc joints) within Neanderthals, early modern and recent modern humans, respectively. By quantifying the shape of all the joints of the TMc complex together, we can holistically characterize its morphology in each species. This characterization can elucidate which specific thumb movements and, by extension, which grip(s) would have been favored by this morphology; that is, would each TMc complex be better suited to precision (i.e., involvement of the pad of the fingers in opposition to the pad of the thumb) or power grips (i.e., involvement of the palm of the hand).

**Table 1:** The fossil sample.

Species	Specimens	Date	Sex	Location	Cultural association	Acquisition methods^c^
Neanderthals	La Ferrassie 1	Middle Paleolithic—43–45 ka^36^	M	France	Mousterian	P
	La Ferrassie 2	Middle Paleolithic—43–45 ka^36^	F	France	Mousterian	P
	Le Régourdou 1^a^	Late Middle Paleolithic—75 ka^37^	I^b^	France	Mousterian	LS
	Shanidar 4^a^	Middle Paleolithic—46–54 ka^38^	M	Iraq	Mousterian	LS
	Kebara 2^a^	Middle Paleolithic—43–50 ka^39^	M	Israel	Mousterian	P
Early modern humans	Qafzeh 9	Middle Paleolithic—95 ka^40^	F	Israel	Mousterian	μCT
	Ohalo II H2	Early Upper Paleolithic—19 ka^41^	M	Israel	Kebaran	μCT
	Abri Pataud 26227 (P1)	Early Upper Paleolithic—26–28 ka^42^	F	France	"Proto-Magdalenian” (Gravettian)	P
	Abri Pataud 26230 (P3)	Early Upper Paleolithic—26–28 ka^42^	F	France	"Proto-Magdalenian” (Gravettian)	P
	Dame du Cavillon	Early upper paleolithic—24 ka^43^	F	France	Gravettian	P

^a^Casts.

^b^I = indeterminate sex, n.b. the sex of Le Régourdou 1 is still debated.

^**c**^*μCT* micro-computed tomography, *LS* laser scanning, *P* photogrammetry.

Following previous studies of external and internal bone morphology, we predict that humans will demonstrate a TMc complex morphology that favors thumb abduction^44–46^ as this movement, combined with axial pronation and flexion of the thumb, comprises thumb opposition. An opposed thumb is habitually used by modern humans in strong precision “pad-to-pad” grips^47^, in which the thumb pad opposes the index finger pad, and the joints of the TMc complex are oriented obliquely relative to the transverse plane. In contrast, we predict that Neanderthals will show a morphology of the TMc complex favoring extended thumb movements, associated with axially/parasagitally-oriented joints. This morphology is consistent with habitual use of a transverse power squeeze grip, in which an object is held transversely across the palm of the hand with strongly flexed fingers and the thumb is extended and adducted to brace against the object^48^. This grip was used by humans when using hafted tools to scrape wood in an experimental setting^49^. Thus, by studying the manner of shape covariation within the TMc complex, we can infer the degree to which Neanderthals and modern humans shared similar biomechanical advantages for high loading from a thumb held in different postures during varied manipulative or tool-related behaviours^3,6–8^.

## Results

A multivariate regression of shape on centroid size tested for the size effects on morphology. No allometric effect was found for either the trapezium or Mc1 indicating that the size of bone alone cannot explain shape differences found between individuals and taxa (Supplementary Information Table S2).

**Figure 2:**
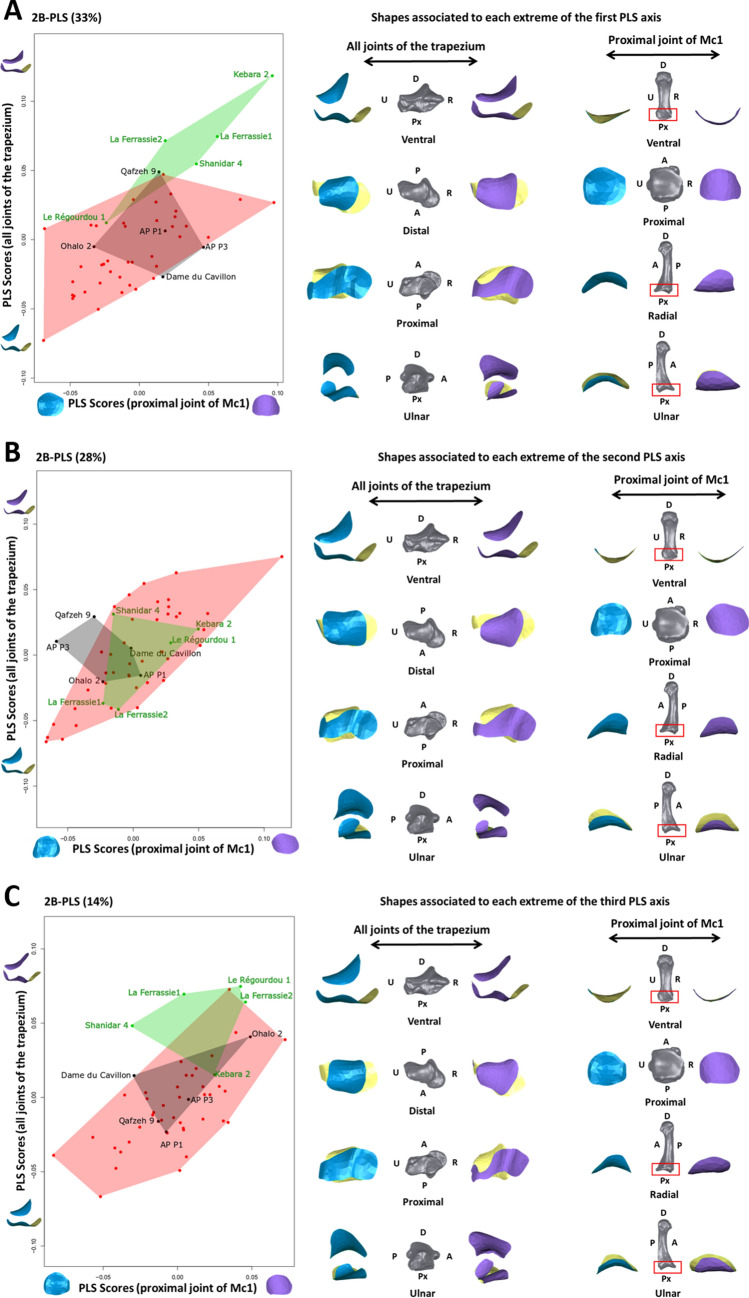
2B-PLS of shape covariation between the proximal joint of Mc1 and all joints of the trapezium across taxa. (**A**) 1st PLS axis; (**B**) 2nd PLS axis; (**C**) 3rd PLS axis. Neanderthals (green), early modern humans (black) and modern humans (red). The figures on the right represent the shapes associated with each minimum and maximum of the shape covariation axes (in blue and purple, respectively) in different anatomical views (the full bone of a random *H. sapiens* individual is depicted with each surface to aid interpretation). All shapes are scaled to approximately the same length. *A* anterior, *P* posterior, *D* distal, *Px* proximal, *R* radial, *U* ulnar.

The 2B-PLS analysis showed that patterns of shape covariation between the joints of trapezium and the Mc1 were significantly different between Neanderthals and recent humans (Fig. 2A,C; Table 2). Early modern humans showed no significant shape covariation differences with either recent humans or Neanderthals (Table 2), and were always placed intermediately in the PLS axes, presenting a shape covariation pattern between recent humans and Neanderthals (Fig. 2). The 2B-PLS analysis revealed substantial intraspecific variation in shape covariation for both recent humans and Neanderthals (Fig. 2).

**Table 2:** Results of omnibus and subsequent pairwise one-way permutational MANOVAs on the first three PLS axes testing for differences in shape covariation between joints of trapezium and proximal joint of the Mc1 across taxa, between the side of the bones (right and left) and sex.

	2B-PLS between all the joints of the trapezium and the Mc1 proximal joint		
	PLS1	PLS2	PLS3
All taxa	**< 0.0001**	0.6409	**0.0028**
Recent modern humans/early modern humans	0.8895	–	1
Recent modern humans/Neanderthals	**0.0006**	–	**0.0012**
Neanderthals/early modern humans	0.1464	–	0.1179
Side of the bones	0.0708	0.5351	0.6055
Sex	0.1404	0.2288	0.8324

Group multivariate variances were not significantly different (*p* > 0.05) and pairwise one-way permutational MANOVAs were only carried out when omnibus permutational MANOVA tests were significant. All values marked in bold where significant at *p* < 0.05, and are reported subsequent to a Bonferroni correction.

**Figure 3:**
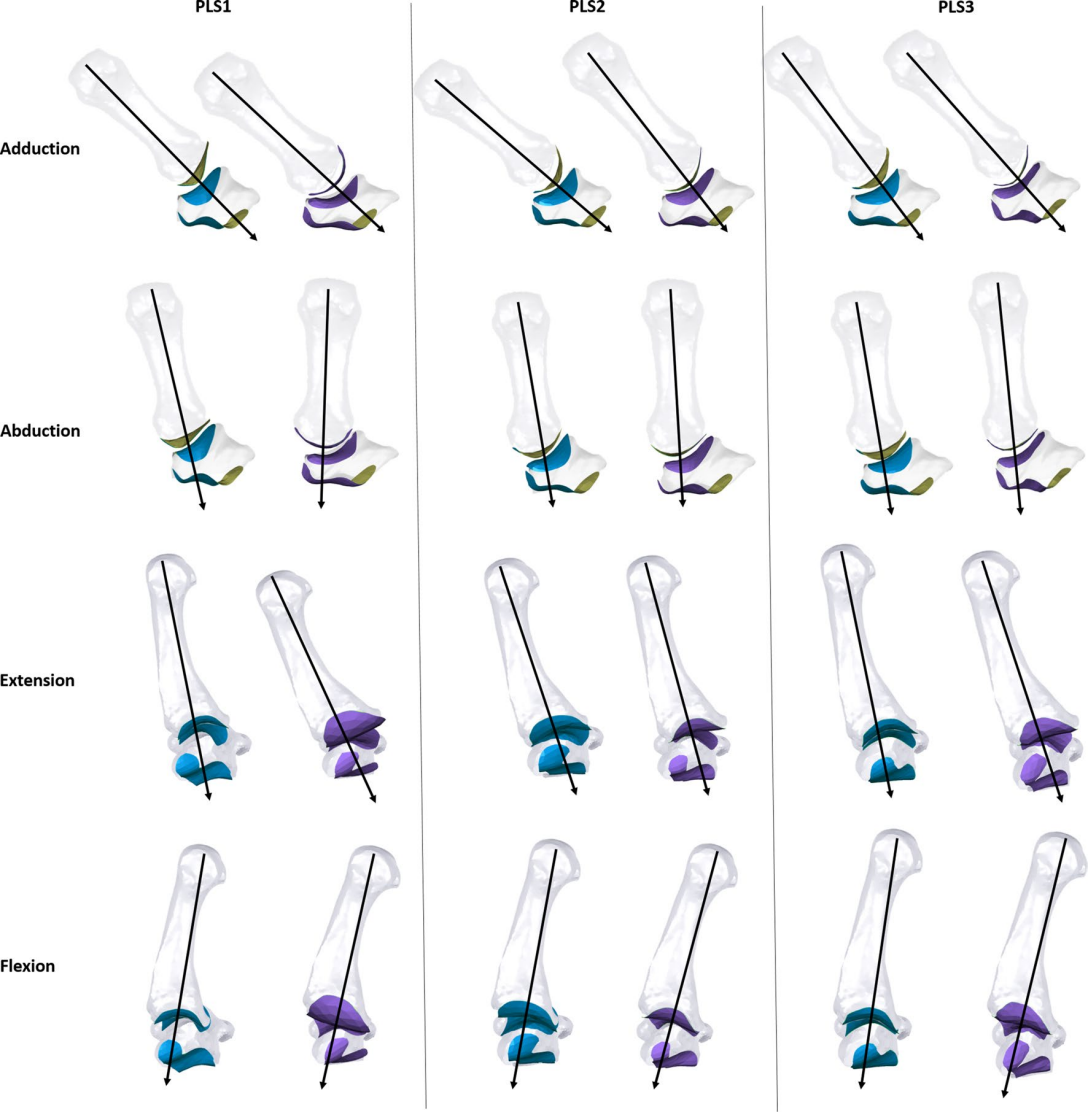
Illustration of possible movements of the TMc complex according to the shape covariations associated with each positive (purple) and negative (blue) extremes of the first—through-third PLS axes. For each shape configuration a direction of force transmission from the Mc1 to the trapezium is suggested (black arrow). The illustration is not scaled.

**Figure 4:**
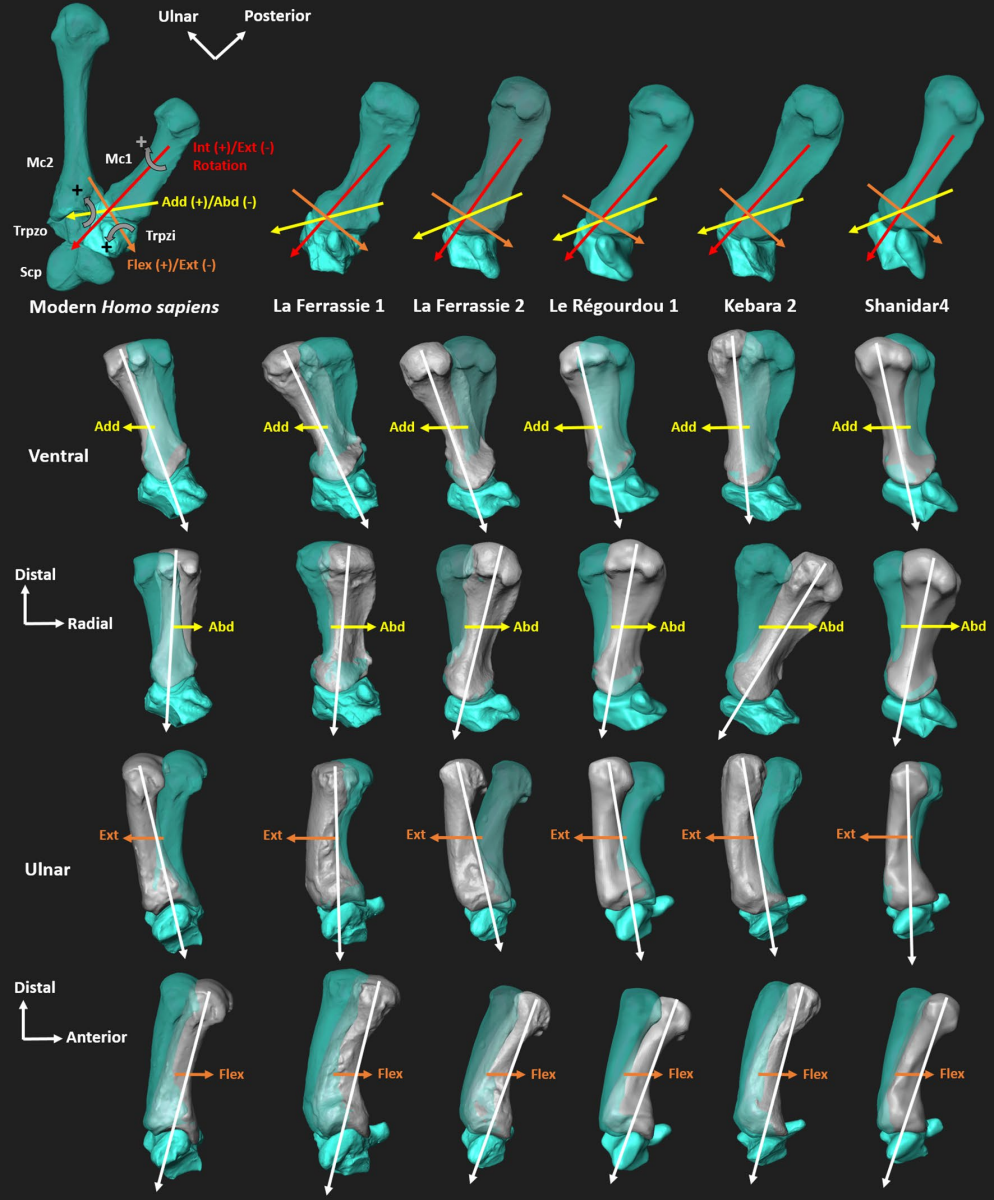
Illustration of potential TMc joint motion in the recent modern human (first column) and for the Neanderthal sample. The modern human specimen lies at the negative extreme end of the first PLS axis (Fig. 3A). This modern human specimen shows the other bones articulation with the trapezium (Trpzi) and the first metacarpal (Mc1), the scaphoid (Scp), trapezoid (Trpzo) and second metacarpal (Mc2). Each column corresponds to the suggested direction of trapezial-Mc1 joint motion (following^50^) for one specimen. The bones are shown in neutral position (turquoise) and in motion (grey). Directions of motion are internal (Int +) and external (Ext −) rotation (red), in adduction (Add +) and abduction (Abd −) (yellow), as well as flexion (Flex +) and extension (Ext −) (orange). For each motion direction of force transmission from the Mc1 to the trapezium is suggested based on the covarying morphology (white arrow). The trapezial-Mc1 joint is surrounded by a strong complex of ligaments and tendons^6,33^, which is not considered in this illustration, as we don’t have these soft tissues for fossils. Rotational movements are not shown here. The illustration is not scaled.

The plot of the first PLS axis (PLS1) (33% of total covariance) separated Neanderthals (positive values on PLS1 axis) from recent humans (negative values on PLS1 axis; Table 2), a difference that was statistically significant. However, the Le Régourdou 1 Neanderthal fell within the recent human morphological range of variation (Fig. 2A), and Qafzeh 9, the oldest early modern human in our sample, fell within the Neanderthal morphological range of variation (Fig. 2A). The range of PLS1 axis values reflected both differences in shape and relative joint orientation, and these features did not vary in the same way within Neanderthals and within modern humans. In recent modern humans (negative values on PLS1 axis), the joint surfaces of both the trapezium and Mc1 were generally more curved and more obliquely-oriented relative to the transverse plane, and the trapezial-Mc1 joint showed an extension of the radial border that would be advantageous for more abducted, rather than adducted, thumb movements (Figs. 2A, 3). In contrast, Neanderthals (positive values on PLS1 axis) showed joint surfaces of both the trapezium and the Mc1 that were flatter and oriented closer to the transverse plane, with the exception of the trapezial-Mc2 joint, which was oriented roughly parasagittally (Figs. 2A, 3). The anteroposterior-flat and radioulnarly-convex shape of the Neanderthal trapezial-Mc1 joint is radioulnarly wider and so more advantageous for a greater range of radio-ulnar, as well as extended, thumb movements compared to recent modern humans (Figs. 2A, 3). Two Neanderthal individuals fell out at opposite extremes (Fig. 2A); Le Régourdou 1 was the only Neanderthal to fall within the modern human range of variation, while Kebara 2 was at the extreme positive side of the axis, being most distinct from modern human shape covariation (Fig. 4).

The plot of PLS2 axis (28% of total covariance) revealed substantial overlap in shape covariation between species, with all Neanderthals and all but two early modern human individuals (Qafzeh 9 and AP-P3) falling within the range of variation seen in recent humans (Fig. 2B). For individuals on the negative side of the PLS2 axis (including Neanderthals specimens, La Ferrassie 1 and 2), the shape covariation was characterized by a posteroulnarly extended articular surface of the trapezial-Mc1 joint, which could be more advantageous for extended and adducted thumb movements. The trapezium joints were more obliquely-oriented relative to the transverse plane, apart from the trapezial-Mc2 joint, which was oriented roughly orthogonal to the transverse plane (Figs. 2B, 3). In contrast, individuals on the positive side of the PLS2 axis (including Neanderthal specimens Kebara 2, Le Régourdou 1, Shanidar 4) showed a posterioradially extended surface of the trapezial-Mc1 joint that could be advantageous for extended and abducted thumb movements, and with joints more transversally-oriented (Figs. 2B, 3).

The plot of the PLS3 axis (14% of total covariance) showed overlap between taxa but Neanderthals (positive values on PLS3 axis) were significantly different from recent humans (negative values on PLS3 axis). Kebara 2 fell near the centre of the recent human distribution and two recent humans fell within the Neanderthal distribution (Fig. 2C; Table 2). The PLS3 axis showed high intraspecific variation in shape covariation of recent humans but also distinguished western European Neanderthals (extreme positive values on PLS3 axis) from Near Eastern Neanderthals, which were closer to the modern human distribution (Fig. 2C). The morphologies reflected by PLS3 axis for western European Neanderthals and one recent human were quite similar to those of the PLS2 axis: a flat and broad trapezial-Mc1 joint associated with an anteroposteriorly thin ulnar portion of the trapezial-trapezoid joint, and joints more obliquely-oriented relative to the transverse plane, apart from the trapezial-Mc2 joint, which was oriented roughly orthogonal to the transverse plane (Fig. 2C). The trapezial-Mc1 joint showed extension of the radial border that could be advantageous for abducted and extended movements of the thumb (Figs. 2C, 3). In contrast, the recent human specimens on the negative side of this axis showed anteroposteriorly broad joints, a more anteroposteriorly-curved trapezial-Mc1 joint obliquely-oriented relative to the transverse plane, a larger trapezial-trapezoid joint, and more transversely-oriented trapezial joints (Fig. 2C). Furthermore, the shape of the trapezial-Mc1 joint showed extension of the radial and ulnar border that would be advantageous for a greater range of radioulnar movements of the thumb (Figs. 2C, 3).

## Discussion

We found significantly different patterns of shape covariation in Neanderthals and modern humans on PLS axes that cumulatively comprise half of the total shape covariation (Fig. 2A,C). These patterns demonstrate different shapes and relative joint orientations that suggest contrasting patterns of habitual thumb movements and force transmission in Neanderthals and modern humans.

The shape covariation patterns in early and recent modern humans support previous studies; most joints are more obliquely-oriented relative to the transverse plane, which suggests a biomechanical adaptation to the transmission of oblique force from the radial side of the hand^3,6–8^. Thus, the general shape covariation pattern of the recent modern human TMc complex would be advantageous for abducted thumb movements that would obliquely load the large trapezial-trapezoid articular surface^6^. This human pattern is therefore also consistent with the habitual use of forceful precision grips involving abduction of the thumb, such as during forceful “pad-to-pad” precision grips^3,47,48^. Interestingly, around half of modern humans have a slightly different TMc complex morphology that could be more advantageous for adducted thumb movements (Figs. 2B, 3), which are used during oblique power squeeze gripping (defined as an object held diagonally across the palm of the hand, clenched by flexed fingers and buttressed by adducted thumb)^48^, and powerful “pad-to-side” grip (handling of objects by the thumb pad and the side of the index finger^3^). These results are consistent with that of Karakostis et al.^16^ that found different hand bone entheseal patterns between individuals known to engage in heavy manual work compared to precision workers. Thus, the variation we found among modern humans may reflect different habitual manual activities across our recent human sample.

In contrast to modern humans, most of the Neanderthals—though their intraspecific variation is high—possess trapezial carpometacarpal joints that are more parallel to the transverse plane while the trapezial-Mc2 joint is oriented parasagittally. Together, the joint orientations of the Neanderthal TMc complex suggest a biomechanical adaptation to the transmission of axial/parasagittal (i.e., parallel to the long axis of Mc1) force from the thumb across the radial side of the hand^3,7,8,14^. The general shape covariation pattern would facilitate an extended and adducted thumb during opposition of the thumb with the other fingers in Neanderthals. This thumb posture suggests the habitual use of powerful transverse power squeeze grips, like those used to grip hafted tools^12,49^. The large axial loads generated by this grip could be distributed across the joint surfaces provided by the more orthogonal/axial orientation of the trapezial-Mc2 and trapezial-scaphoid joints in Neanderthals. The relatively smaller trapezial-trapezoid joint surface on the Neanderthal trapezium also suggests that a greater proportion of Mc1 load would be transmitted to the trapezium and the scaphoid. Conversely, the large size of this joint in humans favours more force transmission across the anterior trapezoid to the capitate during the power grip^6^. This pattern of shape covariation of Neanderthal TMc morphology could have mechanically disadvantaged thumb abduction during grips such as powerful “pad-to-pad” grip involving strong abduction, flexion and rotation of the thumb^3^ since more force would likely be transmitted through the smaller trapezial-trapezoid joint (Figs. 3, 4). However, we do not mean to suggest Neanderthals were incapable of the abducted hand postures, but merely that their morphology made this less mechanically advantageous than in modern humans. Indeed, Karakostis et al.^16^ have shown that the same Neanderthals specimens, apart Le Régourdou 1, possess an entheseal pattern consistent with this type of precision grasping.

We cannot directly associate Neanderthal hand morphology with the specific lithic assemblages as we do not know which individuals, or species in some cases, made or used these artefacts. However, we know that late *Homo* species produced stone tools in this temporal and geographical context. The different lithic technology and typology found, can inform us about behavioural traditions occupying the region. Kebara 2 and Le Régourdou 1 showed the most extreme differences in shape covariation among our Neanderthal sample (Fig. 2A,C). The morphology of the TMc complex of Kebara 2 suggests mechanical advantage when loading a more abducted thumb (Fig. 4), in agreement with current trabecular evidence^51^, suggesting a morphology favoring the use of “pad-to-pad” grips. This result is consistent with that of Karakostis et al.^16^ in which the Kebara 2 entheseal morphology suggests habitual use of precision grips. Also, the Kebara 2 trapezium has a narrow and transversely-oriented Mc2 facet that brings it closer to the ulnar portion of the Mc1 facet. This particular morphology could be disadvantageous to transmitting high load from the Mc2 to the trapezium during the adducted thumb posture of powerful “pad-to-side” grips typically used with short and small flakes^26^. This is consistent with the Mousterian technology at Kebara where there are few retouched flakes^27^ and a greater abundance of longer flakes compared to Le Régourdou 1. Le Régourdou 1 is the only Neanderthal in our sample associated with Quina lithics, an industry with a high proportion of scrapers^37^, and smaller tools than those associated with Kebara 2. Le Régourdou 1 has a morphology advantageous for loading an adducted thumb, that this is used in a “pad-to-side” grips used on scrapers. Therefore though it is only circumstantial evidence, it is interesting that the two most disparate fossil Neanderthals in our sample appear to have morphologies that would be mechanically advantageous for the grips associated with the type of tools frequently found in techno complexes evidenced at the same site where these morphologies were found.

We found no significant differences in shape covariation between early modern humans and Neanderthals, although sample sizes were small. The range of morphological variation found in early modern humans was intermediate between that of recent modern humans and Neanderthals. Interestingly, the closest early modern human to Neanderthals was Qafzeh 9, the oldest individual in our sample at 95 ka^40^ (Fig. 2A). Qafzeh hominins (found in Israel) and Near Eastern Neanderthals existed during the same time period and both were found in association with Middle Paleolithic industry, the Mousterian lithic technologies^40^. However, previous analyses of the Qafzeh 9 hand morphology have interpreted this individual has likely using finer and precise finger movements more often than Neanderthals^11^, suggesting the use of similar technology but with different manual abilities. The other early modern humans in our sample, all younger than Qafzeh 9, were within the recent human range of morphological variation, and are associated with a different technological context (i.e., including more blade tools) than Qafzeh 9^41–43^.

To conclude, our results demonstrate that modern human and Neanderthal TMc complex morphology does not covary in the same manner. Neanderthals possess trapezial carpometacarpal joints that are flatter and more transversely oriented with extension of their radial and ulnar borders, a trapezial-Mc2 joint that is orthogonal relative to the transverse plane, and a small trapezial-trapezoid joint surface. All these features suggest transmission of axial force from the thumb across the radial side of the hand, favoring more extended and adducted thumb movements during powerful opposition of the thumb with the other fingers. In support of shape covariation reflecting habitual hand use, our results show that both Levantine and European Neanderthals in our sample possess a thumb morphology suited for use in transverse power squeeze grips on hafted tools. Although it should be noted that Shea^27^ suggested that Levantine Mousterians could have more frequently utilized hafted artefacts (e.g., spear points) than European Mousterians. The morphology of Neanderthal hands analyzed here, would better facilitate a type of force transmission through the wrist bones associated with the use hafted tools, than that associated with non-hafted tools such as small flakes that require the use of “pad-to-side” or “pad-to-pad” grips^3^. Comparing fossil morphology with contemporaneous lithic industries can help us to infer past behavior and better understand the evolution of modern human manipulative abilities.

## Materials and methods

### Materials

The sample of recent modern humans comprises 40 adults with no sign of external pathology from a broad geographic range (North America, Europe, Africa, North Asia; Supplementary Information Table S1). As the fossil sample of early modern humans and Neanderthals includes individuals of both or unknown sex and bones from both right and left sides, our comparative human sample incorporated the same range of variation: 22 males, 15 females, three with no sex identified, and 25 bones (paired trapezium-Mc1) from the right side and 15 from the left. Original fossils specimens were used for La Ferrassie 1 and 2, and we used high-quality resin casts of the original specimens for Kebara 2, Le Régourdou 1 and Shanidar 4 (see Table 1 for additional information about these fossils). All the data were analyzed together as neither sex nor side significantly affected shape covariation (Table 2).

### 3D scanning

Shape covariation of the Mc1 and trapezium were explored using 3D digital surface models created from scan data collected via different methods including micro-computed tomography (μCT), laser scanning (LS), and photogrammetry (P) (Supplementary Information Table S1). The μCT scans of the samples were obtained as in Stephens et al.^45^. The 3D models from μCT scans were constructed from TIFF data using Avizo 6.3 (FEI Visualization Sciences Group, Hillsboro, USA) software. The LS scans were obtained with a NextEngine laser scanner using a resolution of 28,000 points per square centimeter. Twelve scans were taken at different positions on both side of the bone and then merged using the ScanStudio HD PRO software. P scans were obtained using a Nikon D5100 DSLR camera with a resolution of 24 megapixels with a focal length was fixed to 55 mm (Objectif AF-S DX NIKKOR 18–55 mm VR II) for all pictures. Fifty pictures were captured on both sides of the bone from different viewpoints. For the reconstruction of the 3D models we used the Agisoft PhotoScan software (2014 Agisoft LLC) obtaining a pixel size of 0.00490961 × 0.00490961 mm. Final meshes were created using the Agisoft PhotoScan software with high values of 180,000 optimal number of polygons. Scanning artifacts or anomalies in the polygonal mesh, from all the µCt and LS methods, were corrected using Geomagic Wrap 2015 (3D Systems, Inc) software. All imaging data were analyzed together as there was no significant effect of acquisition method on shape variation across species for either the trapezium joints or the Mc1 joint (MANOVA tests, *p* > 0.05). As we used right and left bones, we mirrored the left bones using Geomagic Wrap 2015 software, in order to ensure homologous comparisons.

### 3D geometric morphometrics

Because of the shape complexity of wrist bones and the challenges of identifying homologous anatomical landmarks (i.e., point locations that are biologically homologous between species) on irregularly-shaped joint surfaces, we quantified shape variation using a GM approach with both 3D anatomical landmarks and 3D sliding semi-landmarks on curves and surfaces^22^. 3D sliding semi-landmarks allow for the accurate description of anatomical zones of high biological interest (like joint surfaces) even if the lack clear anatomical landmarks. 3D sliding semi-landmarks on curves and surfaces correspond to Type III landmarks, in the typology of Bookstein^52^, which are geometric points dependent on the location of other landmarks. Consequently, these semi-landmarks do not constitute absolute anatomical reference points and so additional operations must be performed to be able to use them for shape comparisons (see description of sliding procedure below).

Initially we created a landmark template for each bone by manually placing 3D anatomical landmarks and 3D sliding semi-landmarks on curves and surfaces on one specimen (Fig. 1, and Supplementary Information Figure S1 and Table S3), using Landmark^53^. Type II 3D anatomical landmarks^52^ (five for the trapezium and two for the Mc1) were defined as points of maximum curvature at the limits of joint surfaces on each specimen (described in Supplementary Information Table S3). 3D curves were defined at the margins of articular surfaces and were bordered by anatomical landmarks as recommended by Gunz et al*.*^54^. The curves were digitized with a high density of points (62–142 points per curve depending on the curve length) and then sub-sampled to the number listed in supplementary information (Supplementary Information Table S3). A high density of 3D sliding semi-landmarks were manually placed at approximately equidistant intervals on the entire surface of each bone (147 for all the joints of the trapezium and 41 for the proximal joint of first metacarpal). The template used for the trapezium contains a total of 294 points including five anatomical landmarks, 142 semi-landmarks sliding on curves, and 147 semi-landmarks sliding on surfaces (Fig. 1 and Supplementary Information Figure S1). The template used for the Mc1 contains a total of 105 points including two anatomical landmarks, 62 semi-landmarks sliding on curves and 41 semi-landmarks sliding on surfaces (Fig. 1 and Supplementary Information Figure S1). To assess the repeatability of the manual placement of the anatomical landmarks and curves for the trapezium joints and the Mc1 proximal joint, we landmarked three similar Neanderthal specimens (same sex, side and bone) ten times. Following a procrustes procedure, the first two principle components of principle components analyses (PCA) revealed that shape variation among the repetitions on each specimen tested was much lower than inter-specimen shape variation (Supplementary Information Figure S2). Anatomical landmarks and curves for both bones were thus considered repeatable.

The landmarking procedure continued by manually placing anatomical landmarks and sliding semi-landmarks on curves on all the specimens, as was done for the templates. Next, surface sliding semi-landmarks were projected onto each of the bone’s surface^22^ using the function “placePatch” in the “Morpho” package^55^ in R^56^. Then, the function “relaxLM” in the “Morpho” package was used to relax landmark configuration onto each surface of both bones (Mc1 and trapezium) by minimizing bending energy^55^. A sliding procedure was then performed using the function “slider3d” in the “Morpho” package by minimizing the Procrustes distance (see for details^22,54^). After sliding, a general Procrustes analysis^57^ was performed for each specimen with the function “procSym” in the “Morpho” package, controlling for differences in size, position and orientation of the bones between specimens. After this step, all landmarks and sliding semi-landmarks can be analyzed as Procrustes 3D landmarks. Finally, the size of each specimen, and for each bone separately, was quantified as centroid size (i.e. square root of the summed of squared distances between each landmark and the center of gravity)^52^ in order to test for potentially confounding allometric effects on the study (see below).

### Statistical analysis

To reduce our large data set for statistical analysis, principle components analyses (PCA) were performed using on the Procustes landmark sets using the function “procSym” in “Morpho” package^55^ on R. To investigate patterns of shape covariation between the trapezium and the Mc1, Two-Block Partial Least-Squares (2B-PLS) analyses^58^ were performed on the principle component (PC) scores of each specimen with the “pls2B” function in the Morpho package^55^. By calculating a covariance matrix, 2B-PLS identifies axes that describe common shape variation between the two bones (PLS axes) while reducing dimensionality of the dataset. To visualize the co-varying morphology changes associated with the extremes of each PLS axes, the “plsCoVar” function in “Morpho” was used^55^. To test for differences between the mean shape covariation across the three groups (early modern humans, recent humans and Neanderthals) omnibus one-way permutational MANOVAs (1000 permutations) were run on the Euclidean distance matrices of the first three PLS axes scores (i.e. those that described more than 10% of the total covariance). If these omnibus tests were significant, pairwise versions of the same test were run to understand which groups were significantly different from each other. These permutational MANOVA’s were run using the “Vegan”^59^ and “RVAideMemoire”^60^ packages with the “adonis” and “pairwise.perm.manova” functions, respectively. Before we performed these tests, a test of multivariate homogeneity of variance was performed on the Euclidean distance matrix that describes the PLS scores (function “betadisper” in the “Vegan” package) and a Bonferroni correction was applied to all pairwise results, to ensure valid comparisons (Table 2). Allometric effects on the results were tested using the function “procD.lm” in the “geomorph” package^61^.

## Supplementary information

Supplementary Informations.

## Data Availability

All data needed to evaluate the conclusions in the paper are present in the paper and/or the Supplementary Information. Additional data related to this paper may be requested from the authors.
