# Supplementary material for: The implications of thumb movements for Neanderthal and modern human manipulation
Source: Sci Rep. 2020 Nov 26;10:19323. doi: 10.1038/s41598-020-75694-2 (PMC7692544; doi:10.1038/s41598-020-75694-2)
Supplement: Supplementary file 1 — Supplementary Informations. [file 41598_2020_75694_MOESM1_ESM.pdf]

Supplementary Information for

The implications of thumb movements for Neanderthal and modern human manipulation

Ameline Bardo [1]*, Marie-Hélène Moncel [2], Christopher J. Dunmore [1], Tracy L. Kivell [1,3], Emmanuelle Pouydebat [4], Raphaël Cornette [5]

[1] Skeletal Biology Research Centre, School of Anthropology and Conservation, University of Kent, Canterbury, Kent, CT2 7NR, United Kingdom.

[2] Département Homme et environnement, UMR7194–HNHP (CNRS – MNHN – UPVD – Sorbonne Universités), 1 rue René-Panhard, Paris, 75005, France.

[3] Department of Human Evolution, Max Planck Institute for Evolutionary Anthropology, D-04103 Leipzig, Germany.

[4] UMR 7179 Mécanismes Adaptatifs et Evolution (CNRS, MNHN), Muséum national d'Histoire naturelle, 55 rue Buffon, 75005 Paris, France.

[5] Institut de Systématique, Evolution, Biodiversité (ISYEB), Muséum national d'Histoire naturelle, CNRS, Sorbonne Université, EPHE, Université des Antilles, CP 50, 57 rue Cuvier, 75005 Paris, France.

*Corresponding author. Email: A.Bardo-2183@kent.ac.uk

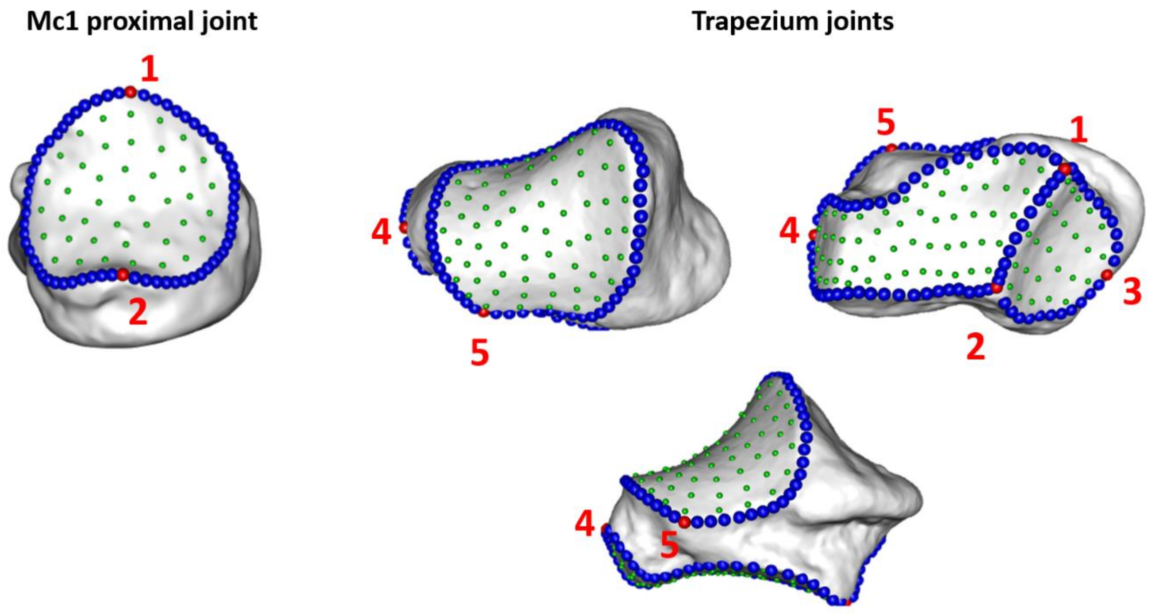

Figure S1. Templates of all the landmarks used in our analyses to quantify shape covariation. Points in red correspond 3D anatomical landmark following the type II of landmarks homology [52]; in blue are 3D semi-landmarks sliding on curves; and in green are 3D sliding semi-landmarks on surface.

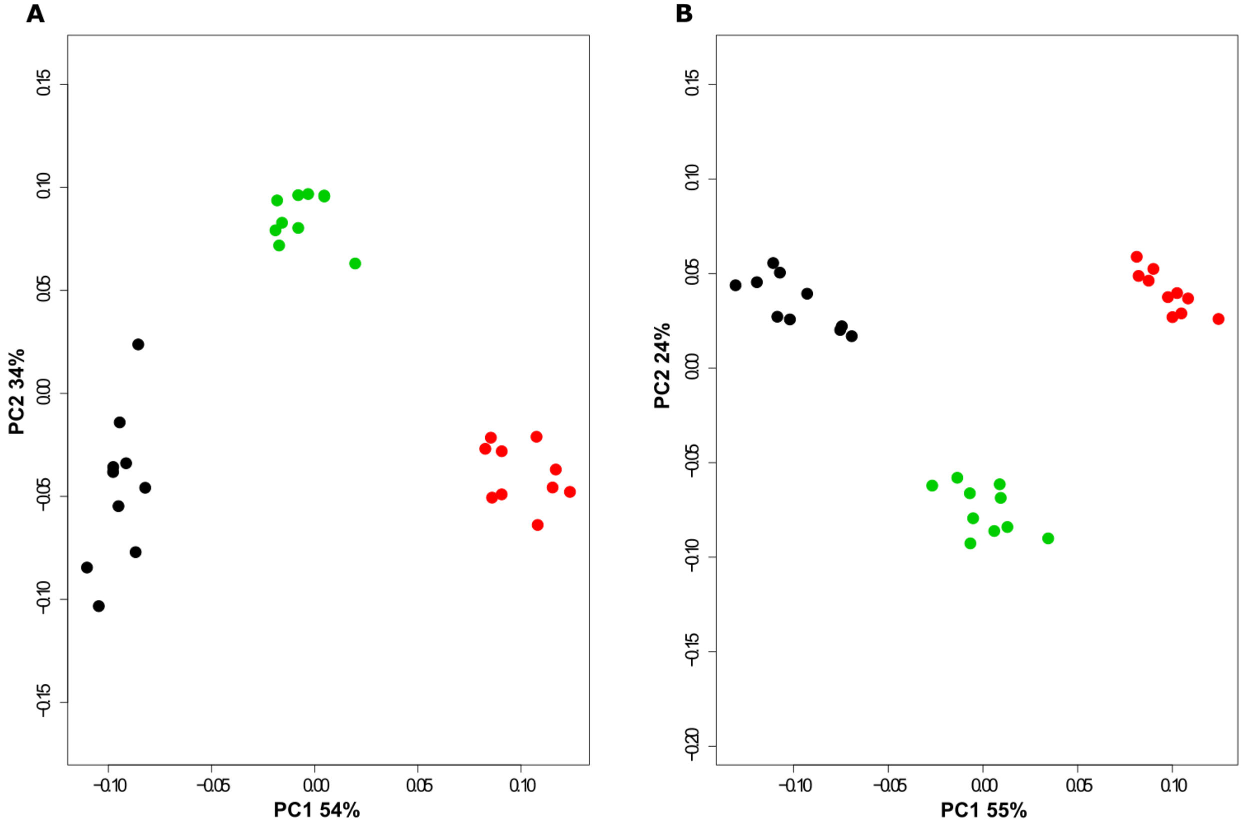

Figure S2. PCAs of anatomical and curve landmark repeatability on the Mc1 (**A**) and trapezium (**B**)**.** Each individual bone was landmarked 10 times on different days. After a Procrustes transformation, PCAs were run. Each colour corresponds to one specimen and each point corresponds to a repetition of landmark placement. The two first PCs (88% of the total variance for the Mc1 and 79% for the trapezium) demonstrate that variation among the landmark repetitions is much lower than that between specimens.

Table S1. Detail of the modern *Homo sapiens* specimens used in analyses. The sample is composed of 40 individuals, including associated hand skeletons of 17 specimens from France[1], four Khoisan rainforest hunter-gatherers from Africa, one from Russia, one from United States, one Nubian Egyptians, eight individuals from Germany, six from United Kingdom and two from Sicily.

Institutions[2]	Inventory number	Sex[3]	Hand(s) side	Native country (Locality)	Acquisition methods[4]
MNHN	MNHN-HA-35047-1	F	R	France	P
MNHN	MNHN-HA-35055-1	F	L	France	P
MNHN	MNHN-HA-35060-1	F	R	France	LS
MNHN	MNHN-HA-35065-1	F	L	France	P
MNHN	MNHN-HA-35068-1	F	L	France	P
MNHN	MNHN-HA-35071-1	F	L	France	P
MNHN	MNHN-HA-35073-1	F	R	France	P
MNHN	MNHN-HA-35080-1	F	L	France	P
MNHN	MNHN-HA-18003	F	R	Russia (Iaroslavl)	P
MNHN	MNHN-HA-10254	F	R	United States (Santa Cruz Island)	P
MNHN	MNHN-HA-18449-1	F	L	Central Africa (Oubangui)	P
MNHN	MNHN-HA-34992-1	M	L	France	P
MNHN	MNHN-HA-34997-1	M	L	France	P
MNHN	MNHN-HA-35003-1	M	L	France	P
MNHN	MNHN-HA-35009	M	L	France	P
MNHN	MNHN-HA-35015-1	M	L	France	P
MNHN	MNHN-HA-35018-1	M	L	France	P
MNHN	MNHN-HA-35026-1	M	L	France	P
MNHN	MNHN-HA-28907	M	R	France	P
MNHN	MNHN-HA-28913-2	M	L	France	P
MNHN	MNHN-HA-17762-2	M	R	Central Africa (near Nola and Bangui)	P
MNHN	MNHN-HA-17980-2	M	R	Congo (Ouésso)	P
MNHN	MNHN-HA-22257-1	M	L	Equatorial Africa	P
GAUG	GAUG-INDEN_91	M	R	Inden, Germany	μCT
GAUG	GAUG-INDEN_117	M	R	Inden, Germany	μCT
GAUG	GAUG-INDEN_118	F	R	Inden, Germany	μCT

GAUG	GAUG-INDEN_119	M	R	Inden, Germany	µCT
GAUG	GAUG-INDEN_243	M	R	Inden, Germany	µCT
GAUG	GAUG-INDEN_311	M	R	Inden, Germany	µCT
GAUG	GAUG-INDEN_319	NA	R	Inden, Germany	µCT
GAUG	GAUG-INDEN_323	NA	R	Inden, Germany	µCT
NHMW	NHMW_J_2	M	R	Nubian Egyptians	µCT
UNIFL	UNIFL_4865	M	R	Syracuse, Sicily	µCT
UNIFL	UNIFL_4887	F	R	Syracuse, Sicily	µCT
KU	NGB_89_SK15_1247	F	R	Canterbury, United Kingdom	µCT
KU	NGB_89_SK15_1250	NA	R	Canterbury, United Kingdom	µCT
KU	NGB_89_SK87	M	R	Canterbury, United Kingdom	µCT
KU	NGA_88_SK-1137	M	R	Canterbury, United Kingdom	µCT
KU	NGA_88_SK-821	M	R	Canterbury, United Kingdom	µCT
KU	NGA_SK_69	F	R	Canterbury, United Kingdom	µCT

[1] *Homo sapiens* specimens who died in Paris (France) are from a collection established by Georges Olivier in the 1950s at the Musée de l'Homme, Paris consisting of unclaimed bodies from Paris hospitals, and so could be people native to another country.

[2] Institutional abbreviations are as follows: MNHN= Muséum National d'Histoire Naturelle, Paris; GAUG Georg-August-Universität Göttingen, Anthropology collection; NHMW = Naturhistorisches Museum Wien; UNIFL = University Florence; KU = Kent University, Canterbury Burials collection.

[3] M= male, F= female, NA= no answer, R= right, L= left

[4] µCT = micro-computed tomography, LS = laser scanning, P = photogrammetry.

Table S2. Results of the multivariate regression of shape on centroid size tested for the size effects on morphology.

	F	df	p.value
Trapezium (all joints)	1.2835	48	0.173
Mc1 (proximal joint)	0.363	48	0.963

Table S3. Definition of the anatomical landmarks, and sliding semi-landmarks, on curves on the trapezium and the Mc1 [15, 46]. Curves are bordered by anatomical landmarks with the first number corresponding of the initial anatomical landmark and the second of the terminal anatomical landmark, or initiated and terminated with the same anatomical landmark.

	Landmark	Definition
**Trapezium**	1	Most anterior aspect of the scaphoid articular surface
	2	Most posterior aspect of the scaphoid articular surface
	3	Most distal aspect of the scaphoid articular surface
	4	Most distal aspect of the Mc2 articular surface
	5	Most ventral aspect of the Point of maximum of curvature of the medial border of the Mc1 articular surface
Curves	1-2, 2-3, 3-1	3 curves to surrounded the scaphoid articular surface
	1-5, 5-2, 1-2	3 curves to surrounded the trapezoid and the Mc2 articular surfaces
	6	1 curve to surrounded the Mc1 articular surface
**Mc1**	1	Most palmar aspect of the proximal articular surface, the ‘tip’ of the palmar beak.
	2	Most dorsal aspect of the articular surface on the metacarpal base.
Curves	1-2	2 curves to surrounded the TMc joint surface
